# Dairy Products and Prevention of Type 2 Diabetes: Implications for Research and Practice

**DOI:** 10.3389/fendo.2013.00090

**Published:** 2013-07-23

**Authors:** Maria Kalergis, Sylvie S. L. Leung Yinko, Roxana Nedelcu

**Affiliations:** ^1^Dairy Farmers of Canada, Montreal, QC, Canada; ^2^Division of Clinical Epidemiology, McGill University Health Centre, Montreal, QC, Canada; ^3^School of Dietetics and Human Nutrition, McGill University, Montreal, QC, Canada

**Keywords:** dairy products, milk, dairy, diabetes, T2D, metabolic syndrome

## Abstract

A growing body of scientific evidence has linked dairy intake to a reduced type 2 diabetes (T2D) risk. Using an evidence-based approach, we reviewed the most recent and strongest evidence on the relationship between dairy intake and the risk of T2D. Evidence indicates that dairy intake is significantly associated with a reduced T2D risk, and likely in a dose-response manner. The association between low-fat dairy and T2D risk reduction appears consistent. A beneficial impact is suggested for regular-fat dairy. The role of specific dairy products needs to be clarified. Potential underlying mechanisms include the role of dairy products in obesity and metabolic syndrome, as well as several dairy components, such as calcium, vitamin D, dairy fat, and specifically *trans*-palmitoleic acid. To conclude, there is strong, consistent, and accumulating evidence that dairy intake reduces the risk of T2D. More research is needed to better understand the role of regular-fat and specific dairy products. Well-designed randomized controlled trials and mechanistic studies are needed to support these findings. Efforts to translate this evidence into clinical practice and public health guidance are needed.

## Introduction

Diabetes is a global epidemic with major health, social, and economic implications. Over the last three decades, the prevalence of diabetes has more than doubled, and more than 371 million people worldwide now have diabetes (1). In 2012, diabetes was the cause of 4.8 million deaths and the estimated health care cost of diabetes was $471 billion worldwide (1).

Type 2 diabetes (T2D) accounts for at least 90% of all diabetes cases and its rates have been increasing at an alarming rate in every country (2). Lifestyle has been identified as playing a major role in the development and prevention of T2D. T2D usually develops during adulthood and is associated with obesity, physical inactivity, and unhealthy diets (2).

A growing body of scientific evidence has linked dairy products to a reduced risk of T2D. Potential underlying mechanisms remain to be fully elucidated but may include a beneficial role of dairy products in obesity and metabolic syndrome (MetS), two important risk factors for T2D, as well as a beneficial role of certain dairy components such as calcium, vitamin D, dairy fat, and specifically *trans*-palmitoleic acid.

Unfortunately, dairy products remain largely under-consumed by all age groups. An astonishing number of Americans (more than 80% men and 90% women) do not meet the minimum dairy requirements of the Dietary Guidelines for Americans (DGA) (3). A large proportion of Canadians also do not meet the minimum daily recommendations established by Health Canada, with an average intake for adults being 1.52 servings, which is well below the 2–4 recommended daily servings (4). Under-consumption of dairy products has also been reported in many other parts of the world (5–7).

Given the increasing amount of evidence on the beneficial role of dairy products in T2D risk reduction, ensuring optimal dairy intake may represent an additional key strategy against this epidemic. In this review, we aim to examine and synthesize the scientific evidence on the association between dairy products and T2D risk, and identify opportunities for future research. Using a comprehensive evidence-based approach, we reviewed the evidence on the relationship between dairy product intake and the risk of T2D. Online databases were searched until March 2013 for relevant peer-reviewed articles. Priority was given to the most recent and strongest available evidence, with a focus on meta-analyses, randomized controlled trials (RCTs), and prospective cohort studies.

## Dairy and Type 2 Diabetes

### Systematic reviews and meta-analyses

Three meta-analyses of prospective cohort studies on dairy products and T2D were identified (Table 1). In a meta-analysis by Tong et al. the highest dairy consumption, compared to the lowest category, significantly reduced the risk of T2D by 14% (combined relative risk (RR) 0.86, 95% confidence interval (CI) 0.79–0.92) (8). A significant inverse association with T2D was also observed for low-fat dairy and yogurt. High-fat dairy and whole (regular-fat) milk were not found to be associated with T2D. A dose-response analysis showed a decrease of 6% in T2D risk per each additional daily serving of total dairy (RR 0.94, 95% CI 0.92–0.97). A dose-response relationship between low-fat dairy consumption and T2D was also present, with a 10% risk reduction per additional serving (RR 0.90, 95% CI 0.85–0.95).

**Table 1:** **List of studies reviewed and main findings on dairy products and T2D**.

Study	Main findings
**SYSTEMATIC REVIEWS AND META-ANALYSES**	
Kratz et al. (20)	Inverse association between high-fat dairy products and T2D
Tong et al. (8)	T2D risk reduction by 14% for highest diary intake
	Significant inverse association for low-fat dairy and yogurt
	No association for high-fat dairy and whole milk
	Decrease of 6% in T2D risk per each additional serving of total dairy per day
	Decrease of 10% in T2D risk per each additional serving of low-fat dairy per day
Elwood et al. (9)	Reduction in diabetes incidence by 4–9% for each additional daily serving of dairy products
Pittas et al. (10)	Lower risk of incident T2D for the highest vs. lowest dairy intake (3–5 vs. 1.5 servings per day)
2010 Dietary Guidelines for Americans	Moderate evidence indicates that dairy products is associated with a reduced risk of T2D
**PROSPECTIVE COHORT STUDIES**	
Grantham et al. (12)	Significant inverse association between the highest tertile of dairy intake and risk of diabetes among men
	Significant inverse association between low-fat milk and diabetes
Louie et al. (13)	No association between total dairy consumption and T2D
	No association for reduced/low-fat dairy and regular-fat dairy
	Significant reduction by 59% in risk of MetS with regular-fat dairy
Struijk et al. (14)	No significant association between total dairy intake and T2D incidence
	Beneficial effect of cheese and fermented dairy on glucose regulation indices
Soedamah-Muthu et al. (15)	No significant association between total dairy consumption or specific types of dairy products and T2D
	Association of fermented dairy products with reduced risk of overall mortality
Fumeron et al. (16)	Inverse association between total consumption of dairy products and impaired fasting glycemia and T2D
	Inverse relationship between cheese and incident metabolic syndrome
Malik et al. (17)	Significantly reduced risk of T2D by 38% for highest quintile of dairy consumption during adolescence (two servings per day)
	A 43% reduction in risk of T2D with consistently high intakes of dairy products (from adolescence to adulthood)
	A 25% risk reduction for the highest current consumption of dairy products (two servings per 1,000 kcal)
Sluijs et al. (18)	No association between total dairy consumption and T2D
	Inverse association between cheese and fermented dairy products and T2D
Mozaffarian et al. (21, 22)	Significant reduction by 48–62% in risk of incident T2D with *trans*-palmitoleic acid
	Direct association between dairy fat and *trans*-palmitoleic acid levels
	Inverse association with risk of T2D for whole (regular-fat) dairy across all intake levels

In their meta-analysis of four prospective cohort studies on diabetes, Elwood et al. demonstrated that milk or dairy consumption was protective against T2D (RR 0.92, 95% CI 0.86–0.97). Each additional daily serving was significantly associated with a reduction in diabetes incidence by 4–9% (9). In another meta-analysis with mostly similar cohort studies, the highest vs. lowest dairy intake (3–5 vs. 1.5 servings per day) was associated with a lower risk of incident T2D [odds ratio (OR) 0.86, 95% CI 0.79–0.93] (10). Furthermore, in the 2010 DGA, the Dietary Guidelines Advisory Committee (DGAC) concluded that moderate evidence indicates that dairy products are associated with a reduced risk of T2D after their systematic review of literature (11).

### Prospective cohort studies

Several prospective cohort studies, published thereafter, have also been conducted to evaluate the potential role of dairy products in T2D prevention (Table 1).

The Australian Diabetes Obesity and Lifestyle Study (AusDiab), a population-based, prospective survey with follow-up of 5 years, consisting of 5,582 adults demonstrated a significant inverse association between the highest tertile of dairy intake and risk of diabetes in men, after adjustment for age, sex, energy intake, and various other clinical characteristics (adjusted OR 0.53, 95% CI 0.29–0.96) (12). Among women, a non-significant inverse association was observed (adjusted OR 0.71, 95% CI 0.48–1.05). Among subtypes of dairy products (low-fat milk, full-fat milk, yogurt, cheese), only low-fat milk had a significant inverse association with diabetes (adjusted OR 0.65, 95% CI 0.44–0.94).

Conversely, in another Australian prospective cohort study, the Blue Mountains Eye Study (BMES), after adjustment for covariates including energy intake, fiber from vegetables, dietary glycemic load, and calcium, total dairy consumption was not found to be associated with T2D (adjusted OR 1.50, 95% CI 0.47–4.77) (13). No association was also observed for reduced/low-fat dairy (adjusted OR 1.09, 95% CI 0.57–2.09), or regular-fat dairy (adjusted OR 0.87, 95% CI 0.48–1.57), and T2D risk. However, compared with subjects in the lowest intake quartile of regular-fat dairy products, those in the highest quartile had a 59% lower risk of MetS (multivariate adjusted OR 0.41, 95% CI 0.23–0.71).

A prospective analysis of the Inter99 Study, a Danish population-based lifestyle intervention, assessed the association between specific types of dairy products and T2D incidence. Glucose regulation, an important underlying mechanism in the development of T2D, was also investigated (14). No significant association was found between total dairy intake and T2D incidence (OR 0.95, 95% CI 0.86–1.06). There was also no association between specific dairy products and T2D, but cheese and fermented dairy appeared to have a beneficial effect on glucose regulation indices. A linear inverse association was demonstrated between cheese and 2 h plasma glucose (β = −0.048, 95% CI −0.095 to −0.001). Fermented dairy products (cheese, yogurt, and buttermilk) were also inversely associated with fasting plasma glucose and hemoglobin A1c.

The Whitehall II prospective cohort study was a London-based study of the working staff of Civil Service departments (15). Ten-year follow-up of 4,186 participants indicated that total dairy consumption was not significantly associated with T2D [hazard ratio (HR) 1.30, 95% CI 0.95–1.77]. Neither high-fat dairy, low-fat dairy, total milk, yogurt, cheese nor fermented products were associated with T2D risk. However, fermented dairy products were significantly associated with an inverse risk of overall mortality.

The Data from the Epidemiological Study on Insulin Resistance Syndrome (DESIR) study was a prospective cohort study of French adults, followed for 9 years (16). Adjustment was made for potential confounders including fat intake, and analysis of 3,435 participants demonstrated that total consumption of dairy products, excluding cheese, was inversely associated with incident impaired fasting glycemia and T2D (adjusted OR 0.85, 95% CI 0.76–0.94). Cheese consumption was not associated with T2D (adjusted OR 0.93, 95% CI 0.82–1.06), but an inverse relationship was found between cheese and incident MetS (adjusted OR 0.82, 95% CI 0.71–0.95).

Data from 37,038 women from the Nurses’ Health Study II, followed for 7 years, was used to evaluate whether dairy consumption during adolescence was associated with the development of T2D in adulthood (17). After adjustment for risk factors present in adolescence, those in the highest quintile of dairy consumption during adolescence (two servings per day) had a reduced risk of T2D by 38%. Adjusting for risk factors present in adulthood also showed a significant inverse association between adolescent dairy intake and T2D (RR 0.73, 95% CI 0.54–0.97). A 43% reduction in risk of T2D was observed for women with consistently high-dairy intakes (from adolescence to adulthood), highlighting the importance of persistence in dairy consumption. A 25% risk reduction was also observed for the highest current dairy consumption (two servings per 1,000 kcal), and a 26 and 28% risk reduction with low- and high-fat dairy, respectively.

In a nested case-cohort analysis within eight countries of the European Prospective Investigation into Cancer and Nutrition (EPIC) Study, consisting of a subcohort of 16,835 participants, total dairy consumption was not associated with T2D (HR 1.01, 95% CI 0.83–1.34) (18). Both the consumption of cheese and fermented dairy products (cheese, yogurt, and thick fermented milk) were inversely associated with T2D (HR 0.88, 95% CI 0.76–1.02 and HR 0.88, 95% CI 0.78–0.99, respectively).

### Randomized controlled trials

To our knowledge, no RCTs to date have specifically assessed the association between dairy products and the risk of incident T2D. However, in a randomized crossover trial of 12 months, the consumption of low-fat dairy (four servings per day) was associated with improved insulin resistance, without adversely affecting body weight and lipid status (19).

### Regular/high-fat dairy

While the evidence appears to be relatively consistent with respect to a beneficial role of low-fat dairy products in the prevention of T2D, the role of regular/high-fat dairy, as well as dairy fat itself, is less clear. In a recent systematic review of observational studies (20), the majority of studies suggested that high-fat dairy products were inversely associated with T2D, either significantly or non-significantly.

In a prospective cohort analysis of the Cardiovascular Health Study (CHS), *trans*-palmitoleic acid, a naturally occurring *trans* fatty acid found in dairy and ruminant fat, was shown to significantly and considerably reduce the risk of incident T2D, with a risk reduction of 62% (21). Dairy fat content appeared to be directly related to *trans*-palmitoleic acid levels, and whole-fat but not low-fat dairy, was also inversely associated with the risk of T2D. In another subsequent prospective cohort study, the Multi-Ethnic Study of Atherosclerosis (MESA), *trans*-palmitoleic acid was associated with a 48% lower risk of incident T2D (22). A pooled meta-analysis of these two independent cohorts indicated a 29% lower incidence of diabetes for each 0.05% point of higher *trans*-palmitoleic acid concentrations (22).

### Specific types of dairy products

The evidence regarding the role of specific types of dairy products such as milk, yogurt, and cheese is limited. Milk has generally been assessed as part of total dairy consumption, and limited evidence exists on milk specifically. It appears that milk consumption may be associated with a reduced risk of T2D, with no association for regular-fat or whole milk and T2D (8, 9, 15). Limited evidence suggests that yogurt is inversely associated with T2D (8, 12, 14). Cheese consumption may be associated with a reduced risk of T2D, but this needs to be confirmed as some findings are not statistically significant (14–16, 18). Finally, a protective role of fermented dairy products as a whole (including yogurt, cheese, buttermilk, and fermented milk), is suggested against T2D (14, 18).

## Potential Mechanisms

### Obesity and weight

Including dairy products in weight loss diets has been shown to be favorable by decreasing adiposity, while preserving lean mass. In a meta-analysis of RCTs, high-dairy calorie-restricted diets led to a significantly greater reduction in body weight, waist circumference, and fat mass, while increasing lean mass significantly more than conventional weight loss diets (23). In another meta-analysis, dairy consumption, in the short-term, coupled with calorie-restriction, had a beneficial impact on body fat reduction (24). Two systematic reviews of observational studies also showed that increased dairy consumption may be protective against weight gain (20, 25).

A large prospective analysis of more than 120,000 individuals followed for 12–20 years assessed the role of diet (including dairy) and lifestyle on long-term weight gain and found yogurt to be a factor associated with a beneficial impact on weight (26). This study found no association between high-or-low-fat dairy categories or cheese and weight gain and all liquids, except milk, were associated with weight gain.

The potential mechanisms by which dairy may be protective against weight gain include reduced lipogenesis and increased lipolysis. A high calcium intake may lead to the formation of insoluble soaps by calcium and the prevention of fat absorption (24, 27, 28). As well, a systematic review of RCTs has confirmed a role of calcium in fat oxidation (29). Whey protein may also play a role in muscle sparing and lipid metabolism (30, 31).

### Metabolic syndrome

There is increasing evidence indicating that dairy products may reduce the risk of MetS including a systematic review of observational studies (32). The mechanism appears to be via the effects of different dairy components on specific MetS risk factors, such as blood glucose level and hypertension, amongst others (32).

### Dairy components

As indicated in Table 2, key dairy components, including calcium, vitamin D, dairy fat, and *trans*-palmitoleic acid, have been suggested to contribute to the metabolic pathways that may be implicated in the prevention of T2D.

**Table 2:** **Potential mechanisms of dairy products in preventing type 2 diabetes**.

Obesity and weight	Favorable effect of dairy products during weight loss through fat mass reduction and preservation/augmentation of lean mass
	Reduction of lipogenesis and increase in lipolysis by high calcium intake
	Prevention of fat absorption via the formation of calcium insoluble soaps
	Increased fat oxidation by calcium
	Role of whey protein in muscle sparing and lipid metabolism
Metabolic syndrome	Reduced risk of MetS via improvement of several cardiometabolic risk factors (e.g., blood lipids, blood pressure, abdominal fat)
**DAIRY COMPONENTS**	
Calcium	Regulation of insulin-mediated intracellular processes in insulin-responsive tissues
	Secretory function of pancreatic β-cells
	Phosphorylation of insulin receptors
	Down-regulation of regulator genes encoding pro-inflammatory cytokines involved in insulin resistance
Vitamin D	Direct effect on insulin secretion by binding to vitamin D receptors in pancreatic β-cells
	Indirect effect via the regulation of extracellular calcium
	Protection against systemic inflammation by counteracting cytokine generation
*Trans*-palmitoleic acid	Improvement of hepatic and peripheral insulin resistance, metabolic regulation, and suppression of hepatic *de novo* lipogenesis
	Lower levels of triglycerides, fasting insulin, blood pressure, and C-reactive protein

### Calcium

Calcium may play a key role in regulating insulin-mediated intracellular processes in insulin-responsive tissues (10, 33, 34). Insulin secretion is a calcium-dependent process, and changes in Ca^2+^ flux can adversely affect the secretory function of pancreatic β-cells (10). The phosphorylation of insulin receptors is also a calcium-dependent process (10, 35). Changes in intracellular calcium levels modulate adipocyte metabolism, which may suppress insulin-mediated lipolysis and promote *de novo* lipogenesis, thus leading to fat accumulation (10, 36).

### Vitamin D

Circulating active vitamin D may have a direct effect on insulin secretion by binding to vitamin D receptors in pancreatic β-cells. Vitamin D also has an indirect effect via the regulation of extracellular calcium for normal calcium flux through cell membranes and adequate Ca^2+^ pool (37). Vitamin D may enhance insulin receptor expression and insulin responsiveness for glucose transport (38). Moreover, vitamin D directly activates peroxisome proliferator activator receptor-δ, a transcription factor involved in fatty acid metabolism (39, 40).

T2D has also been shown to be associated with systemic inflammation (41, 42). Insulin sensitivity and β-cell survival may be improved through the direct effect of vitamin D on the generation of cytokines (10). Vitamin D also down-regulates genes encoding pro-inflammatory cytokines involved in insulin resistance (43). The anti-inflammatory mechanism may also be modulated by the regulation of Ca^2+^ levels (10).

### Dairy fat and *trans*-palmitoleic acid

The *trans* isomer of palmitoleic acid (*trans*-16:1n-7) is a natural source of palmitoleic acid, mainly obtained via the diet from dairy or ruminant fat. *Trans*-palmitoleic acid may have similar actions to that of endogenous *cis*-palmitoleic acid (22, 26). Up-regulation of adipose-derived *cis*-palmitoleic acid improves hepatic and peripheral insulin resistance, diminishes metabolic dysregulation, and suppresses hepatic *de novo* lipogenesis (20, 22, 44). Higher *trans*-palimitoleic acids levels are also associated with lower triglycerides, fasting insulin, blood pressure, and C-reactive protein (21, 22).

Other dairy-derived fatty acids have also been associated with lower T2D risk but findings are less consistent (22). A recent meta-analysis of cohort studies has indicated that dietary saturated fat is not associated with risk of T2D (45).

### Limitations

Our review of the most recent studies on dairy products and risk of T2D has some limitations. Most of the studies included in this review consisted of prospective cohort studies. The potential for bias in such observational studies is present in that individuals who have a regular consumption of dairy products may also engage in other healthful diet and exercise behavior. In most studies, adjustments were made for potential confounders, yet, the possibility of residual confounding cannot be completely eliminated.

## Conclusion

There is a strong and relatively consistent body of accumulating evidence indicating that dairy products may significantly reduce the risk of T2D and likely in a dose-response manner. The protective effect of low-fat dairy products against T2D appears consistent in the literature. Evidence on regular-fat dairy products suggests no association with T2D or a beneficial impact. The role of specific dairy products such as cheese and yogurt appears beneficial.

### Implications for future research

More research is needed to better understand the role of regular-fat and specific types of dairy products on incidence of T2D and indices of glycemic regulation. Large RCTs are needed to confirm findings on the role of dairy in the management of cardiometabolic risk factors, particularly in at risk population, such as those with prediabetes.

Randomized controlled trials are also warranted to increase our understanding of the role of individual dairy nutrients *per se*. Moreover, mechanistic studies are essential to understand the underlying mechanisms regarding dairy products and specific dairy components in preventing T2D.

### Implications for practice

Given the evidence regarding dairy products in T2D prevention, translation into practice, and clinical and public health guidance is warranted. The value of having an adequate intake of dairy products is to be reinforced especially during weight loss and among those with prediabetes and MetS.

Identifying adequate dairy intake as a key strategy in clinical practice guidelines for T2D is also needed. Finally, communicating the importance of meeting dairy recommendations should be an essential part of public health guidance and identifying strategies to increase dairy consumption to optimal levels is of utmost importance.

## Conflict of Interest Statement

Maria Kalergis is employed at Dairy Farmers of Canada, a non-profit agri-food organization. Sylvie S. L. Leung Yinko and Roxana Nedelcu have consulted for Dairy Farmers of Canada.
